# Nicotine Ingestion Reduces Heart Rate Variability in Young Healthy Adults

**DOI:** 10.1155/2022/4286621

**Published:** 2022-01-04

**Authors:** Qian-nan Guo, Jing Wang, Hong-yan Liu, Dong Wu, Shi-xiu Liao

**Affiliations:** ^1^Henan Provincial People's Hospital, Henan Provincial Key Laboratory of Genetic Diseases and Functional Genomics, National Health Commission Key Laboratory of Birth Defects Prevention, Medical Genetic Institute of Henan Province, People's Hospital of Zhengzhou University, People's Hospital of Henan University, Zhengzhou 450003, China; ^2^Henan College of Finance and Taxation, Zhengzhou 450003, China

## Abstract

Around the whole world, smoking is considered harmful to human health, such as increasing the risk of cardiovascular disease (CVD, such as coronary heart disease and stroke) and lung cancer. The purpose of this study was to explore whether nicotine, the main component of tobacco, has adverse effects on heart rate variability (HRV) in adolescents, so as to remind adolescents not to smoke and not to take pleasure in abusing nicotine. In this study, 40 male and 40 female young healthy nonsmoking subjects were selected to analyze the changes of HRV after taking 4 mg nicotine orally. We found that nicotine reduced HRV in young healthy male and female subjects, and there was no gender difference in this effect (*P* > 0.05). In conclusion, smoking is harmful to the cardiac system of young people, especially when nicotine content ≥4 mg dosage.

## 1. Introduction

Smoking is an important and well-established risk factor for cardiovascular disease. Nicotine is a colourless, poisonous alkaloid, derived from the tobacco plant. It is the substance in tobacco to which smoker can become addicted. Although nicotine is thought to be harmful to human's health, people still use nicotine replacement therapy (NRT), especially Nicabate CQ lozenge, for smoking cessation as it is effective in quitting smoking. The response of nicotine is fast and excitatory in nature. The intake of nicotine will produce similar effects as smoking that increases the sympathetic activity to cause rapid heartbeat, increased blood pressure, rapid shallow breathing, hyperglycemia, and increased level of bad cholesterol-LDL [1].

Heart rate variability (HRV) is the variation of time interval from beat to beat as a result of cardiac autonomic modulation (CAM). The imbalance in CAM is thought to be the causation of heart disease, and HRV measurement is widely accepted to reflect the cardiac autonomic modulation. HRV measurement [2] is well-established for predicting the fitness of a person [3] and used as an indicator for heart risk prediction or monitoring of heart disease progression [4–6]. Many researches have shown that people who were long-term smokers [7] or with coronary heart disease, diabetic neuropathy, or congestive heart failure had low HRV compared with healthy nonsmokers [4, 5]. Therefore, HRV measurement is a fine indicator for expressing the adverse effect of nicotine on heart.

In order to determine whether nicotine has an effect on HRV, and whether this effect is gender dependent, 80 young healthy students (40 males and 40 females, aged 18-22 yrs) were recruited in this research.

## 2. Materials and Methods

### 2.1. Drugs

Treatment: Nicabate CQ lozenge (GlaxoSmithKline) contains nicotine at 4 mg dosage and administered orally.

Placebo: mint lozenge and ingested orally.

### 2.2. Subject

80 young healthy nonsmoking Chinese Han volunteers from Henan College of Finance and Taxation with native place of Henan Province, 40 males (19.48 ± 1.52 yrs, 57.50 ± 5.09 kg, 19.24 ± 1.28 kg/ms^2^) and 40 age, weight, and BMI matched females (19.87 ± 1.47 yrs, 55.14 ± 5.97 kg, 20.48 ± 1.70 kg/ms^2^) aged between 18 and 22 yrs, weighted 42-65 kg, with body mass index (BMI) within 18-23.5 kg/ms^2^, were recruited (there was no any significant difference between females and males at age, weight, and BMI, *P* > 0.05). All these adolescents had never suffered from cardiovascular disease, no implanted cardiac pacemaker, no hypertension (systolic pressure > 140 mmHg or diastolic pressure > 90 mmHg), no hypotension (systolic pressure < 110 mmHg or diastolic pressure < 60 mmHg), no diabetes, no allergy to nicotine, no any medication during or a week before the experiment, and avoid the female if she was in the menstrual period during the experiment.

### 2.3. Method of HRV Measurement and HRV Calculation

In this study, HRV was measured by a short-term (4-minutes) frequency domain method. This method measured the underlying variance (power) [8, 9] in the heart rate pattern at different frequencies (from 0.00 to 0.50 Hz) during the 4-minute ECG measurement. HRV was evaluated as RR interval variance, and the RR interval was determined as the interval between the two R points of the consecutive QRS complex of ECG. Short-term ECG recording for spectral analysis produced three frequency domains: high-frequency (HF: 0.15-0.50 Hz), low-frequency (LF: 0.04-0.15 Hz), and very low-frequency (VLF: 0.00-0.04 Hz) domains. The power (ms^2^) of each domain and the total power of the three domains was recorded. LF power mainly represented sympathetic nervous system (SNS) activity and HF power mainly represented parasympathetic nervous system (PNS) activity [9]. VLF represented neither SNS nor PNS activity and was always be avoided when interpreting the analysis of HRV during a short-term ECG recording. Increased LF and decreased HF indicates the reduced HRV.

Due to individual differences, the total power of RR interval variance was different for each subject. LF power and HF power are part of the total power, so they cannot be directly used for analysis. Therefore, the LF power and HF power were normalized into LF (n.u.) and HF (n.u.) for analysis. LF (n.u.) and HF (n.u.) were referred to as HF and LF hereinafter (Table 1).

### 2.4. Major Equipment

OMRON T8 Automatic Blood Pressure Monitor: measuring HR, systolic, and diastolic blood pressure.

PowerLab 4/25 (AD Instruments): detecting and processing the ECG signal and transferring the ECG signal to the computer for viewing and analysis. Subjects sat in a relaxed upright posture; the ECG was detected by AD instruments using 3 electrodes applied to the body of the subject (the three electrodes were arranged in a line that cross the heart: white electrode sticked to the skin at the right collarbone, black electrode sticked to the skin at the second left rib from the bottom of the ribcage, and the green electrode sticked to the skin near the stomach that was next to the black electrode).

Chart 5 for Windows (AD Instruments, Germany): processing ECG signal and frequency-domain analysis (VLF, LF, and HF).

### 2.5. Procedure

**Figure 1:**
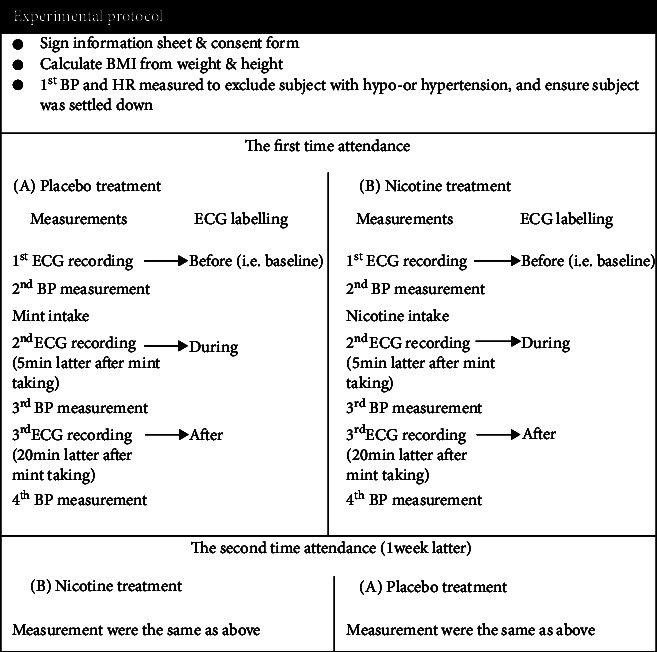
Experimental protocol. The mint was the placebo, and the nicotine was the treatment. All the ECG were recorded in a 4-minute interval.

The examiners and the volunteers were both blind of the order in which the drugs were given or eaten. Every subject was asked to take either nicotine or mint tablet randomly in the first time attendance of the experiment from 15:30 pm to 17:30 pm; then, the other kind of drug was taken 1 week latter at the same time of the day. On the day of the experiment, each subject was asked to not eating and drinking except pure water after 7:30 am (Figure 1) and no alcohol drinking at least three days ago.

Before starting the measurement, the information sheet and the consent form were signed; blood pressure and HR were measured. The height and weight were measured to calculate the BMI (body mass (kg)/height (m)^2^), subject with BMI out of range 18.5-23.5 kg/ms^2^ excluded from this study; the initial blood pressure was measured to exclude subject who had hypertension or hypotension; the initial HR was measured to ensure the subject was settled down.

The ECG was measured at a 4-minute interval, and blood pressure was measured after each ECG measurement section to ensure there was no hypotension or hypertension occurred during the experimental process. Three times of ECG were recorded: before nicotine intake, 10 min after nicotine intake, and 20 min after nicotine intake.

### 2.6. Statistical Methods (Using Excel)

For analysis of mint or nicotine effect on HRV during the absorption period of mint or nicotine: paired *T*-test was performed to compare several HRV measurements between the three trials (before, during, and after, see Figure 1) within the same gender.

To see if there were any gender differences in nicotine effect: two-tail unpaired *T* test was performed to compare male with female for HRV changes from the baseline (experimental trail: before) to during and after.

Calculation of % change in HF (nu), LF (nu), and HR: for an example of subject *X*, % change of his or her HR at experimental trail After = (HR_After_–HR_Before_)/HR_Before_, where HR_Before_ is the value of HR recorded at experimental trail before, i.e., the baseline of HR. % change of HF and LF was calculated in the same way.

Data in the result part was presented as mean ± SEM (standard error of mean, SEM). Significance was chosen at *P* < 0.05.

## 3. Results

### 3.1. Mint (Placebo) Ingestion (See Table 2)

LF (nu) analysis, in female: LF varied from 64.26 ± 2.81 at before to 65.68 ± 2.75 at during and to 63.78 ± 2.67 at after; there was no significant difference between any two of the three experimental trails (*P* > 0.05). In male: LF varied from 64.63 ± 2.27 at before to 65.34 ± 2.54 at during and to 64.28 ± 2.04 at after; there was no any significant difference between any two of the three experimental trails (*P* > 0.05). Therefore, SNS activity was not altered by mint ingestion (Table 2).

HF (nu) analysis, in female: HF varied from 27.87 ± 2.56 at before to 27.31 ± 2.13 at during and to 28.14 ± 2.35 at after; there was no significant difference between any two of the three experiment trails (*P* > 0.05). In male: HF varied from 28.11 ± 2.23 at before to 27.89 ± 2.33 at during and to 28.01 ± 1.82 at after; there was no any significant difference between any two of the three experiment trails (*P* > 0.05). Therefore, PNS activity was not altered by mint ingestion (Table 2).

LF/HF, in female: LF/HF varied from 2.97 ± 0.46 at before to 3.09 ± 0.43 at during and to 2.92 ± 0.41 at after; there was no significant difference between any two of the three experimental trails (*P* > 0.05). In male: LF/HF varied from 2.68 ± 0.30 at before to 2.72 ± 0.31 at during and to 2.66 ± 0.28 at after; there was no any significant difference between any two of the three experimental trails (*P* > 0.05). Therefore, the CAM did not significantly changed upon the mint ingestion in both females and males.

From the above results, they indicated that mint had no obvious effect on HRV in both males and females.

### 3.2. Nicotine Treatment (See Tables 3 and 4)

LF (nu) analysis, in female: LF increased from 65.36 ± 2.61 at before to 67.06 ± 2.75 at during and to 69.95 ± 2.67 at after; there was only a statistically significant difference at after compared with before (*P* < 0.05) (Table 3). In male: LF increased from 65.63 ± 2.27 at before to 67.34 ± 2.54 at during and to 70.35 ± 2.04 at after; there was only a statistically significant difference at after compared with before (*P* < 0.05). Therefore, SNS activity was enhanced by nicotine ingestion and was significantly increased at 20 min after nicotine intake.

HF (nu) analysis, in female: HF decreased from 27.19 ± 2.3 at before to 25.4 ± 2.13 at during and to 23.9 ± 2.35 at after; there was only a statistically significant difference at after compared with before (*P* < 0.05) (Table 3). In male: HF decreased from 28.51 ± 2.23 at before to 26.54 ± 2.33 at during and to 24.32 ± 1.82 at after; there was only a statistically significant difference at after compared with before (*P* < 0.05). Therefore, PNS activity was suppressed by nicotine ingestion, and it was significantly reduced at 20 min after nicotine intake.

LF/HF analysis, in female: LF/HF varied from 3.09 ± 0.47 at before to 3.23 ± 0.40 at during and to 3.86 ± 0.54 at after; there was only a statistically significant difference between experimental trails after and before (*P* < 0.05). In male: LF/HF varied from 2.67 ± 0.27 at before to 3.10 ± 0.38 at during and to 3.44 ± 0.40 at after; there was only a statistically significant difference between experimental trails after and before (*P* < 0.05). Therefore, the CAM was shifted to a SNS predominant manner after nicotine ingestion at 20 min after nicotine intake in both females and males.

HR analysis, in female: HR increased from 78.06 ± 2.69 at before to 82.02 ± 2.92 at during and to 80.51 ± 2.86 at after; there were statistically significant differences at both during and after compared with before (*P* < 0.05). In male: HR increased from 72.65 ± 2.02 at before to 74.48 ± 2.27 at during and to 75.42 ± 2.25 at after; there was only a statistically significant difference at after compared with before (*P* < 0.05). Therefore, nicotine ingestion had significant effect on elevating HR in both male and females, especially at 20 min after nicotine intake.

SDNN analysis, in female: SDNN varied from 71.21 ± 5.12 at before to 72.98 ± 6.08 at during and to 72.58 ± 5.77 at after; there was no significant difference between any two of the three experiment trails (*P* > 0.05). In male: SDNN varied from 93.24 ± 6.50 at before to 86.18 ± 7.05 at during and to 86.01 ± 5.87 at after; there was no significant difference between any two of the three experiment trails (*P* > 0.05). Therefore, nicotine ingestion had no effect on SDNN within 20 min after nicotine intake.

RMSSD analysis, in male: RMSSD varied from 58.64 ± 5.70 at before to 57.69 ± 7.27 at during and to 52.38 ± 5.25 at after; there was a statistically significant difference between experimental trails after and before (*P* < 0.05). In female: RMSSD varied from 46.73 ± 5.34 at before to 46.59 ± 5.84 at during and to 44.74 ± 5.60 at after; there was no statistically significant difference between any two of the three experimental trails (*P* > 0.05). Therefore, nicotine ingestion had significant effect on reducing male RMSSD, which indicated that the PNS activity of CAM was significantly decreased upon nicotine ingestion in males.

% change in LF (nu), HF (nu), and HR: there was no any significant difference in % change between male and female in LF (nu), HF (nu), and HR from before to during and to after upon 4 mg nicotine ingestion (*P* > 0.05, Table 4). Therefore, modulation of CAM activity by nicotine on HRV was not gender different or gender dependent.

Overall, from the above results of LF (nu), HF (nu), LF/HF, HR, and % change in LF (nu) and HF (nu) and HR, they shown that nicotine had effects in reducing HRV, where the CAM activity was shifted to SNS predominant manner rather than PNS, and these effects were not gender different or gender dependent.

### 3.3. Baseline Analysis between Females and Males

LF (nu), HF (nu), LF/HF, RMSSD, and HR were not statistically significantly different between males and females, while SDNN in male was 93.24 ± 6.50 which was statistically significant higher than female (71.21 ± 5.12, *P* < 0.05) (Table 5).

## 4. Discussion

Nicotine can enter the body in many ways, such as smoke inhalation, ingestion, transdermal patch, and intranasal spray. In human, nicotine can easily pass through the blood brain barrier and enter the brain after it enters the human blood circulation, and it takes just 10 to 20 seconds to get nicotine from smoke into the brain. Nicotine has been found to have cognitive-enhancing effects [10, 11] and can help to reduce body weight or appetite [12]. However, the adverse effects of nicotine were much more than its benefits. In human, nicotine is significantly associated with cardiovascular disease, lung cancer, adverse pregnant outcomes, chronic dermatoses [13], senescence, and atherosclerosis [14]. Especially during pregnancy, maternal smoking is causally associated with a number of adverse pregnancy outcomes, including ectopic pregnancy, fetal growth restriction, preterm birth, placental abruption, and orofacial cleft defects [15, 16]. In mice models, maternal nicotine exposure could significantly decrease the weight of both maternal and offspring mice (~30%), inhibit organ growth, inhibit mitochondrial respiration activity, increase the risk of fetal infection in offspring mice (*P* < 0.05), and cause the development of metabolic associated fatty liver disease in adulthood offspring mice [17, 18].

In mammals, nicotine binds with nicotinic acetylcholine receptors (nAChRs). nAChRs are composed of five subunits arranged around a water filled pore [19] and mainly expressed on neurons or axon terminals of neurons. nAChRs mainly includes three types, GABAA, glycine, and serotonin receptors. Therefore, the main role of nAChRs is to regulate the release of neurotransmitters including excitatory neurotransmitter glutamate, inhibitory transmitter GABA, and reward related neurotransmitter dopamine. Thus, nicotine can regulate the function or action of brain, organ, or muscle through its binding with nAChRs located within the central nervous system and the peripheral nervous system.

In the heart, nAChRs are expressed on cardiac myocytes. Nicotine binds to cardiac nAChRs will lead to the activation of cardiac nAChRs, resulting in CAM imbalance. CAM imbalance, characterized by a hyperactive SNS and a hypoactive PNS, is associated with various pathological conditions. Over time, excessive energy demands on the system can lead to cardiovascular disease. HRV can be used to assess CAM imbalance; therefore, the risk of cardiovascular disease is commonly found in people with smoking.

Many researches have done earlier in smoking subjects found that NRT in smoking cessation have no significant disturbances of autonomic cardiac control (including heart rate, systolic blood pressure, hematologic and coagulation indices, and CO level) when compared with smoking event in smokers [20–23]. However, this did not mean NRT is safe. In smokers, NRT still has autonomic effects greater than placebo patch [21]. Several case reports [24–27] still generated concern about NRT safety and suggested a possible causal relationship between NRT and cardiovascular risk. Although NRT is much safer than smoking [28], nicotine is toxic in its nature, a strong and deadly poison in animals. Moreover, the subjects recruited in those studies about NRT effect on cardiovascular system were smokers rather than healthy subjects. Therefore, these experimental results can only draw the conclusion that NRT may not be as bad as smoking, but cannot draw the conclusion that NRT is safe. In order to determine whether NRT is safe, investigation should be done in healthy nonsmokers.

In this experiment, nicotine effect was investigated in young healthy nonsmokers rather than smokers, the nicotine drug used here was Nicabate CQ lozenge (one of NRT) which contained nicotine at 4 mg dosage. Nicotine at 4 mg dosage was thought to be sufficient to cause an obvious effect despite the weight differences of subjects, because 4 mg nicotine was sufficient for smoking cessation in high-dependence smokers with a big range of weight (~76 ± 18 kg) [29]. In addition, there was no significant difference in body weight between male and female subjects recruited in this study; thus, the HRV analysis bias caused by body weight difference could be ignored.

LF measurement mainly represents SNS activity, and HF measurement mainly represents PNS activity. Therefore, in this experiment, the results clearly shown that nicotine indeed has an adverse effect on HRV in both healthy males and females (LF increased, HF decreased) and leads to the transfer of CAM to SNS predominated control (LF/HF increased). The nicotine effect on CAM is rapid as the significance in LF, HF, LF/HF, and HR was observed at only 20 min after nicotine ingestion. Therefore, this experimental results shown that nicotine does have an adverse effect on HRV. HRV is the indicator for predicting the fitness of a person [3] and used as an indicator for heart risk prediction or monitoring of heart disease progression [4]. Therefore, this experimental finding can strongly deny that NRT is safe in human. In conclusion, NRT may not have the same adverse effects as smoking, but NRT is still harmful to people's health.

Overall, nicotine is harmful to cardiac system, and smoking or nicotine replacement therapy (NRT), especially nicotine containment at 4 mg dosage, is not safe for Chinese young adults.

## Figures and Tables

**Table 1:** Definition of the measurements involved in this study.

Name	Unit	Definition
LF power	ms^2^	Power in low frequency range (mainly represents SNS activity)
HF power	ms^2^	Power in high frequency range (mainly represents PNS activity)
LF (nu)	n.u.	LF in normalized units: LF/(LF power + HF power)∗100
HF (nu)	n.u.	HF in normalized units: HF/(LF power + HF power)∗100
BMI	Kg/ms^2^	Body mass index: body mass (kg)/height (m)^2^
HR	BPM	Heart rate
RMSSD	ms	The square root of the mean of the sum of the squares of differences between adjacent NN intervals
SDNN	ms	Standard deviation of all NN intervals

^∗^The LF (nu) and HF (nu) were assessed for HRV analysis in this experiment.

**Table 2:** Mint (placebo) treatment.

Measurements	Mint treatment		
	Before	During	After
LF_F_	64.26 ± 2.81	65.68 ± 2.75	63.78 ± 2.67
LF_M_	64.93 ± 2.27	65.34 ± 2.54	64.28 ± 2.04
HF_F_	27.87 ± 2.56	27.31 ± 2.31	28.14 ± 2.35
HF_M_	28.11 ± 2.23	27.89 ± 2.33	28.01 ± 1.82
LF_F_/HF_F_	2.97 ± 0.46	3.09 ± 0.43	2.92 ± 0.41
LF_M_/HF_M_	2.68 ± 0.30	2.72 ± 0.31	2.66 ± 0.28

Note: data was presented as mean ± SEM; “F” meant female, “M” meant male; LF and HF measurements were in normalized unit.

**Table 3:** Nicotine treatment.

Measurements	Nicotine treatment				
	Before	During	After	*P* ^∗^ (during vs. before)	*P*# (after vs. before)
LF_F_ power (ms^2^)	1999.29 ± 199.13	1972.13 ± 236.72	2206.73 ± 252.97		
LF_M_ power (ms^2^)	2960.90 ± 359.88	3000.17 ± 397.88	2916.57 ± 253.11		
HF_F_ power (ms^2^)	973.15 ± 136.53	916.27 ± 176.27	887.53 ± 162.62		
HF_M_ power (ms^2^)	1389.68 ± 181.02	1396.85 ± 225.55	1110.72 ± 146.94		*P* < 0.05
LF_F_ (n.u.)	65.36 ± 2.61	67.06 ± 2.75	69.95 ± 2.67		*P* < 0.05
LF_M_ (n.u.)	65.63 ± 2.27	67.34 ± 2.54	70.35 ± 2.04		*P* < 0.05
HF_F_ (n.u.)	27.19 ± 2.30	25.40 ± 2.13	23.90 ± 2.35		*P* < 0.05
HF_M_ (n.u.)	28.51 ± 2.23	26.54 ± 2.33	24.32 ± 1.82		*P* < 0.05
LF_F_/HF_F_	3.09 ± 0.47	3.23 ± 0.40	3.86 ± 0.54		*P* < 0.05
LF_M_/HF_M_	2.67 ± 0.27	3.10 ± 0.38	3.44 ± 0.40		*P* < 0.05
HR_F_ (BPM)	78.06 ± 2.69	82.02 ± 2.92	80.51 ± 2.86	*P* < 0.05	*P* < 0.05
HR_M_ (BPM)	72.65 ± 2.02	74.48 ± 2.27	75.42 ± 2.25		*P* < 0.05
SDNN_F_ (ms)	71.21 ± 5.12	72.98 ± 6.08	72.58 ± 5.77		
SDNN_M_ (ms)	93.24 ± 6.50	86.18 ± 7.05	86.01 ± 5.87		
RMSSD_F_ (ms)	46.73 ± 5.34	46.59 ± 5.84	44.74 ± 5.60		*P* = 0.08
RMSSD_M_ (ms)	58.64 ± 5.70	57.69 ± 7.27	52.38 ± 5.25		*P* < 0.05

Note: data was presented as mean ± SEM; “F” meant female, “M” meant male; LF and HF measurements were in normalized unit; only *P* value < 0.05 was annotated.

**Table 4:** Female vs. male for % changes in LF and HF from baseline (before) at during and after 4 mg nicotine ingestion.

Measurements	During		After	
	Female	Male	Female	Male
% change in LF from baseline	3.20 ± 2.97%	3.57 ± 3.39%	7.94 ± 3.13%	13.83 ± 3.09%
	*P* > 0.05		*P* > 0.05	

% change in HF from baseline	2.31 ± 5.96%	4.259 ± 7.47%	11.36 ± 4.47%	12.34 ± 4.99%
	*P* > 0.05		*P* > 0.05	

% change in HR from baseline	5.12 ± 1.27%	2.645 ± 1.789	3.09 ± 0.80	12.34 ± 3.84
	*P* > 0.05		*P* > 0.05	

Note: LF and HF measurements were in normalized unit; % change was presented as mean ± SEM; *P* was the *P* value for female vs. male.

**Table 5:** Female vs. male for HR, LF, HF, LF/HF, SDNN, and RMSSD at baseline.

Measurements	Nicotine treatment					
	LF	HF	LF/HF	SDNN	RMSSD	HR
Baseline	*P* > 0.05	*P* > 0.05	*P* > 0.05	*P* < 0.05	*P* > 0.05	*P* > 0.05

## Data Availability

The data used to support the findings of this study are available from the corresponding author upon request, email: guoqn 1984@aliyun.com.
